# Identification and use of an alkane transporter plug-in for applications in biocatalysis and whole-cell biosensing of alkanes

**DOI:** 10.1038/srep05844

**Published:** 2014-07-28

**Authors:** Chris Grant, Dawid Deszcz, Yu-Chia Wei, Rubéns Julio Martínez-Torres, Phattaraporn Morris, Thomas Folliard, Rakesh Sreenivasan, John Ward, Paul Dalby, John M. Woodley, Frank Baganz

**Affiliations:** 1 Dept. of Biochemical Engineering, Advanced Centre for Biochemical Engineering, University College London, Torrington Place, London WC1E 7JE, U.K; 2 Dept. of Structural and Molecular Biology, ISMB, University College London, Gower Street, London WC1E 6BT, U.K; 3 Department of Chemical and Biochemical Engineering, Technical University of Denmark, DK 2800 Lyngby, Denmark

## Abstract

Effective application of whole-cell devices in synthetic biology and biocatalysis will always require consideration of the uptake of molecules of interest into the cell. Here we demonstrate that the AlkL protein from *Pseudomonas putida* GPo1 is an alkane import protein capable of industrially relevant rates of uptake of C_7_-C_16_ n-alkanes. Without alkL expression, native *E.coli* n-alkane uptake was the rate-limiting step in both the whole-cell bioconversion of C_7_-C_16_ n-alkanes and in the activation of a whole-cell alkane biosensor by C_10_ and C_11_ alkanes. By coexpression of alkL as a transporter plug-in, specific yields improved by up to 100-fold for bioxidation of >C_12_ alkanes to fatty alcohols and acids. The alkL protein was shown to be toxic to the host when overexpressed but when expressed from a vector capable of controlled induction, yields of alkane oxidation were improved a further 10-fold (8 g/L and 1.7 g/g of total oxidized products). Further testing of activity on n-octane with the controlled expression vector revealed the highest reported rates of 120 μmol/min/g and 1 g/L/h total oxidized products. This is the first time AlkL has been shown to directly facilitate enhanced uptake of C_10_-C_16_ alkanes and represents the highest reported gain in product yields resulting from its use.

Using synthetic biology for pathway engineering will require enzymes to be used *in-vivo* rather than isolated or purified. This is particularly true for complex enzymes such as the alkane mono-oxygenase (AlkB) of *Pseudomonas putida* GPo1 which is an integral membrane protein, requiring two coenzymes (AlkG and AlkT) as well as two cofactors (NADH and FAD). Screening the substrate specificity of such enzymes in this context will rely on the assumption that transport of the substrate into the cell occurs at an appropriate rate. The alkane degradation pathway native to *Pseudomonas putida* GPo1 has been the focus of extensive research over the past decades and much is understood about the components of the pathway, the substrate range^1^,^2^, enzyme mechanism^3^, electron transport coenzymes^4^,^5^,^6^,^7^, regulatory system^8^,^9^,^10^, effect on host cell physiology^11^,^12^,^13^,^14^, performance in recombinant hosts^15^,^16^,^17^,^18^ and potential applications^19^. It has previously been hypothesised^20^,^21^,^22^ from the presence of *alkL* in the *alk* operon and its position in the outer membrane^23^ that it plays an important role in transport of alkanes into the cell. Despite this, the role of the *alkL* component in alkane transport has remained unproven with no apparent loss of function observed when *alkL* is knocked out^23^. The authors of the work speculated that the reason no loss of activity was observed is because *alkL* may only be necessary for uptake when growing in conditions where the alkane concentration available is extremely low and therefore diffusion across the membrane alone will not be sufficient. Redundancy of outer membrane transporters with the same function has also been speculated as a possible explanation^21^.

The scientific literature indicates that whilst both the purified alkane hydroxylase complex and the wild-type containing the *alk* genes were able to utilize aliphatic alkanes in the range of C_6_ –C_12_, the recombinant *P.putida S81 pGEc41*, which contains all alk genes with the exception of *alkJ* (an alcohol dehydrogenase), *alkK* (an acyl CoA synthetase) and *alkL*, is not capable of oxidizing n-dodecane^1^,^16^. Use of the *E.coli* pGEc47ΔJ strain, which contains all alk genes except for *alkJ*, has been shown to be capable of n-dodecane bio-oxidation^24^. Although other explanations are available, such as increased metabolism of the product or other intrinsic host properties in the case of the *P.putida* host, the presence of *alkL* on the plasmid is a notable difference between the two strains and therefore in this study its role in uptake of medium chain alkanes is investigated. Recent work by others has also identified that AlkL can enhance oxidation of nonane, methyl dodecanoate^25^ and limonene^26^ in resting cells which further supports the hypothesis for the role of *alkL* in the uptake of medium-chain alkanes.

## Results

### Factorial experimental design for systematically identifying the function of *alkL*

**Figure 1:**
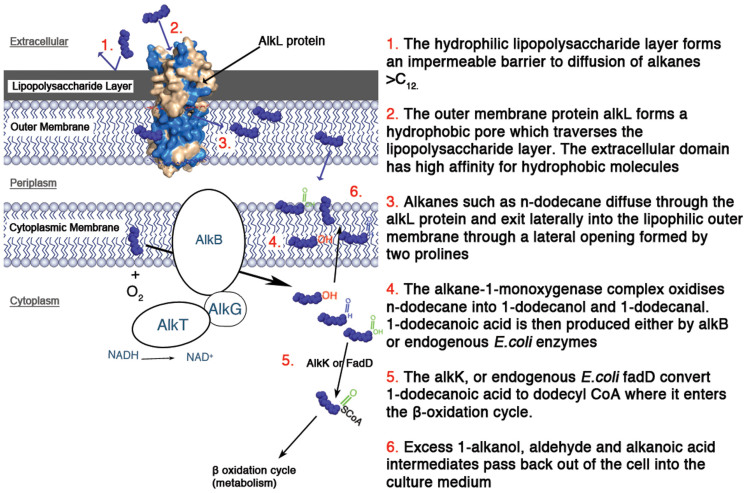
Schematic of proposed mechanism for alkane uptake and bioconversion via the AlkB monooxygenase complex.

To test the hypothesis that *alkL* is necessary for uptake of C_12_ alkanes, *E.coli* expressing the alkane hydroxylase complex of *P.putida GPo1* was used as a test system to determine if uptake was occurring. The *alkL* gene was expressed under control of the pALK promoter on a plasmid containing the alkB,F,G,T genes, which code for the alkane-1-monooxygenase (AlkB), rubredoxin (AlkG) and rubredoxin reductase (AlkT) which form the alkane hydroxylase complex responsible for the oxidation of n-alkanes to 1-alkanols (Figure 1). The regulatory protein AlkS was also present to control expression of the alk genes. An equivalent plasmid without the *alkL* gene was used as a negative control. Previous attempts to oxidise dodecane to 1-dodecanol have identified overoxidation and metabolism of 1-dodecanol as a bottleneck with the pGEc47ΔJ plasmid^24^ and so the plasmid for the new biocatalyst was also designed to minimize metabolism by removal [from the pGEc47ΔJ plasmid] of the aldehyde dehydrogenase (*alkH*) and acyl CoA synthetase (*alkK*) which will contribute to the overoxidation of 1-dodecanol to dodecanoic acid and further into the β-oxidation pathway.

The Clontech in-fusion system^27^ was used for generating these new plasmids containing the minimal set of genes for alkane oxidation in a one-step multi-fragment recombination reaction.

**Figure 2:**
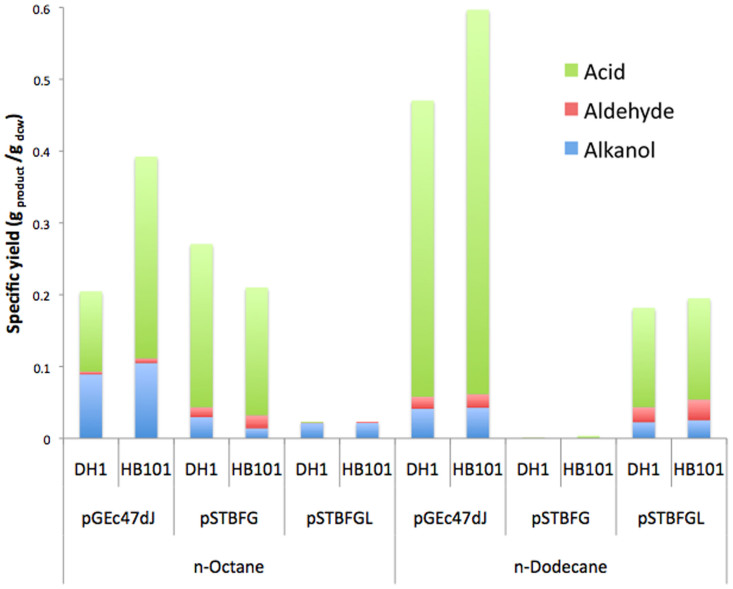
Specific yields (grams of product per gram of dry cell mass) of the alcohol, aldehyde and fatty acid products resulting from bio-oxidation of n-octane and n-dodecane using the pGEC47ΔJ, pSTBFG and pSTBFGL plasmids transformed into *E.coli* DH1 and HB101 according to the factorial design in table S1. The graphs show the average of microwell bioconversions from 8 separate colonies under each condition.

The plasmids were tested using a factorial design in DH1 and HB101 strains of *E.coli* according to Table S1. The results show (Figure 2) that from 16 biological replicates of n-dodecane oxidation using the *E.coli* pSTBFG cells (without the *alkL* gene product), only two showed detectable levels of oxidised product, with yields of 0.004 and 0.006 g_oxidised products_/g_dcw_ after 20 hours, the other 14 showed no detectable product. The host cells containing AlkL showed activity in all 16 replicates on n-dodecane with a maximum yield of 0.73 g_oxidised products_/g_dcw_ (average 0.18 g_oxidised products_/g_dcw_). Oxidised products of n-octane were detected in all host cells containing either pSTBFG or pSTBFGL. This demonstrated that an effective alkane hydroxylase complex was being expressed and was functional in the hosts lacking *alkL* and therefore demonstrating that the presence of *alkL* is necessary for activity on n-dodecane.

The factorial screening experiment revealed that activity of the STBFGL plasmid was achieved over all conditions tested upon n-dodecane. Analysis of variance (ANOVA) was performed on the data and confirmed that the cooperative effect between the plasmid in combination with the substrate (C_8_ or C_12_ alkane) was validated over all conditions and was the most significant of the factors varied (p < 0.001).

SDS-PAGE analysis was performed to confirm *alkB* expression. AlkL levels were too low to detect over the background using SDS-PAGE but its expression was demonstrated by Western blot (Figure S1) and correlated with whole-cell bioxidation activity on dodecane. The work also demonstrates that *alkL* is not strictly necessary for activity on n-octane in *E.coli* although the work of others has shown *alkL* to improve initial rates of n-octane oxyfunctionalisation by 4-fold^25^. In this case, the less hydrophobic n-octane substrate may be able to diffuse across the membrane without a transporter or may be able to be transported into the cell by native transporters in *E.coli*, in particular FadL or OmpW. It seems that effective transport of the longer chain alkanes (C_12_-C_16_) does not occur through these proteins. This may be due to the selectivity of the transporters or that: if these transporters do enable longer chain alkane transport they may do so at insufficient levels for detectable activity with the alkB complex, or alternatively these native transporters may not be adequately expressed. *E.coli* strains expressing alkL were also tested for a direct measurement of n-dodecane uptake via resuspending the cells to a constant cell density, incubation with the alkane substrate for 15 minutes, centrifugation and washing of the *E.coli* pellet twice, extracting the intracellular alkanes into ethyl acetate overnight and quantifying by gas chromatography. The results of this showed that the control cells not expressing alkL bound up to 20% of the alkane compared to 40% in the cells expressing alkL (figure S4).

### Removal of alkH and alkK to reduce overoxidation of 1-alkanols to fatty aldehydes and fatty acids

We have previously reported that overoxidation of the desired 1-dodecanol product to the 1-dodecanal and dodecanoic acid products is a major issue when using the pGEc47ΔJ strain^24^. Since the phenomenon of excessive acid build-up in the absence of the alcohol dehydrogenase *alkJ* was witnessed it was considered that removal of the downstream aldehyde dehydrogenase, *alkH*, would provide information on whether *alkH* was primarily responsible for the conversion from alkanol to aldehyde to acid.

**Figure 3:**
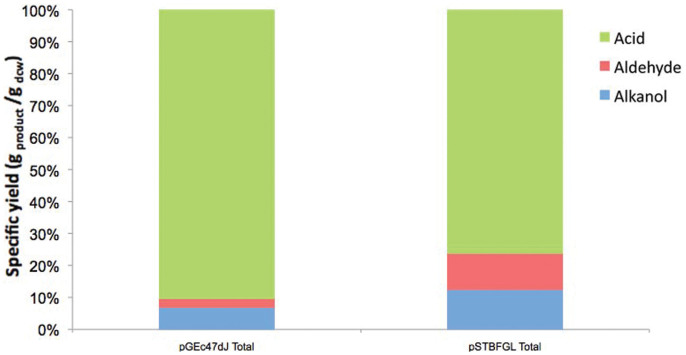
Proportion of 1-dodecanol, 1-dodecanal and dodecanoic acid products as a percentage of the total quantity of oxidized products for bio-oxidation of n-dodecane using the pGEC47ΔJ and pSTBFGL plasmids transformed into *E.coli* DH1 and HB101 according to the factorial design in table S1. The graphs show the average of microwell bioconversions from 16 separate colonies with each plasmid.

Removal of *alkK* and *alkH* from the plasmid resulted in producing a greater average proportion of alkanol and aldehyde products and reducing overoxidation to the carboxylic acid product in the case of n-dodecane (Figure 3). Aldehyde proportion increases from 2% of oxidized products to 10% in the *ΔalkH* strain. An increase in aldehyde yield was expected, however, dodecanoic acid is still the majority product (77% of total oxidized products). This hints that AlkH was not the primary cause of carboxylic acid formation in the pGEc47ΔJ strain. It also provides evidence that AlkB may be responsible for carboxylic acid formation directly. This has been shown to occur in other oxygenase systems such as P450ALK of *Candida tropicalis* which is capable of producing dicarboxylic acids directly from alkane substrates^28^. The isolated alkB enzyme is already known to overoxidise to the aldehyde^2^,^29^ but it has yet to be proven that it is capable of carboxylic acid formation. Although there is evidence linking it to a component under control of the alk promoter in pGEc47ΔJ^24^ and the work reported in this study further supports the hypothesis that AlkB may be capable of oxidation to the carboxylic acid product. However, endogenous *E. coli* enzymes or non-enzymatic oxidation of the unstable aldehyde could also explain this overoxidation or contribute to it.

### Effect of alkB and alkL expression on plasmid stability

The specific activity demonstrated by the new pSTBFG and pSTBFGL constructs was lower than that of the pGEc47ΔJ plasmid for both octane and dodecane substrates. The cell growth was also lower, particularly in the cells containing the pSTBFGL construct (Figure S2), indicating a particularly detrimental effect on cell viability of expressing the alkL protein. There is much evidence in literature of poor stability of alk gene expression^13^,^17^,^30^ with instability in expressing the alkane hydroxylase enzyme (AlkB) observed in both the *P.putida* wild-type host^13^ and in *E.coli* strains W3110 and DH1^17^. SDS-PAGE demonstrated that the AlkB component was being expressed to levels at least as high as the pGEc47ΔJ cells (Figure S3). This indicates that the inferior performance of the new constructs may be caused by negative consequences of overexpression from the high copy number plasmid. This may be as a result of excessive quantities of the electron transport coenzymes AlkG and AlkT (although not seen on the SDS PAGE gel over the background) inclusion body formation of one or more of the Alk components, inadequate translocation of the AlkB protein to the inner membrane, inappropriate ratios of the AlkB, AlkF and AlkG components, compromising the integrity of the membrane or some other limitation. A previous study into this enzyme complex also indicated that overexpression of the complex in W3110 leads to compromised stability and does not result in higher specific activities^6^.

### Controlled expression of the alkL protein to overcome toxicity and control intracellular enzyme activity via transport protein expression level

**Figure 4:**
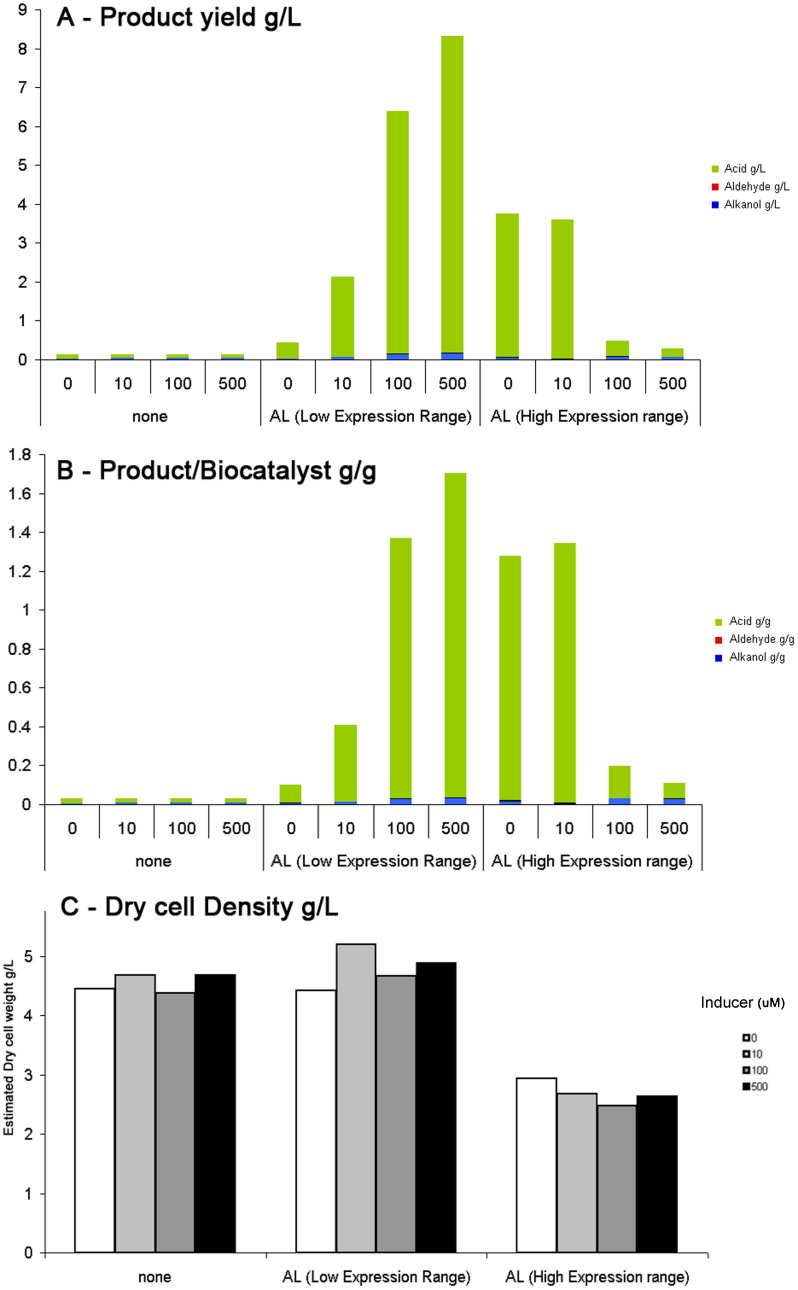
Effect of alkL expression level on volumetric (A) and specific (B) product yields and biomass concentration (C). Graphs A&B show the change in oxidation products of the n-dodecane bio-oxidation using alkB, alkG and alkT from the pGEc41 plasmid by co-expressing alkL using either a low-range or high range expression plasmid under different inducer concentrations. The Y-axis shows the yields of products measured after 24 hours compared to the negative control with only the pGEc41 plasmid. The numbers on the x-axis represent inducer concentrations in μM of Rhamnose.

To overcome the issue of plasmid stability caused by alkL, the alkL protein was cloned into a rhamnose inducible plasmid which allowed tighter control of expression. The plasmid was then co-transformed alongside the pGEC41 plasmid (which lacks alkL) and using this configuration yields of up to 8 g/L of total oxidation products were observed after 24 hours (1.7 g/g) albeit with a greater proportion of dodecanoic acid compared to 1-dodecanol (Figure 4).

The alkL protein was expressed from two different plasmids with different levels of expression. Using the low-range (weak rbs) expression vector showed that n-dodecane oxidation activity could be directly correlated with expression level and that the maximum activities achieved by the un-induced high-range (strong rbs) expression vector could be matched. By changing the ribosome binding site (RBS) to contain an increased spacer region between the RBS and the start codon it was possible to introduce this fine level of rheostatic control of expression by modulating the concentration of rhamnose inducer. This demonstrates a powerful approach which can be applied to optimizing the substrate feeding rate into the cell by modulating the transport characteristics of the cell. Furthermore the overall product yields of dodecanoic acid were twice that of the construct with the original RBS. The specific yield of product (g/g) was equivalent for the two variants but higher cell densities were able to be reached with the modified RBS thus indicating that this strategy seems to be effective to overcome the toxic burden of overexpression of the transport protein as well as finely controlling the the expression level of the transport protein (Figure 4).

### Alkane substrate range

**Figure 5:**
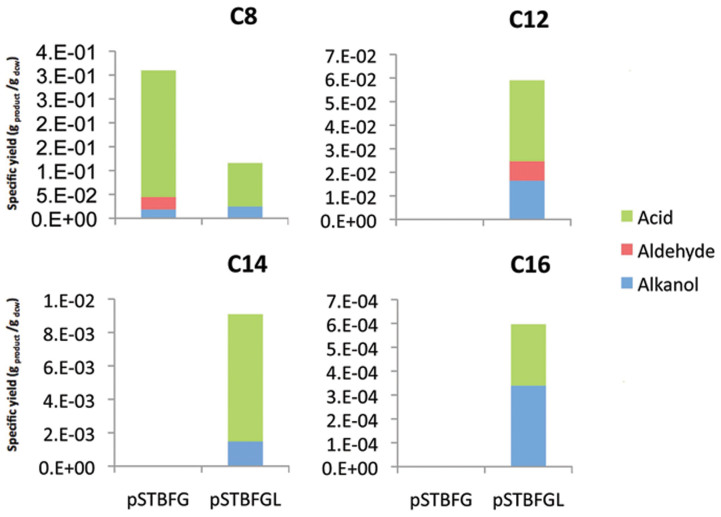
Average specific yield of products per gram of dry cell weight resulting from bio-oxidation of linear alkanes of carbon chain length C_8_- C_16_ (n = 4) cultured according to the conditions stated in the material and methods section.

Now that the role of AlkL in alkane transport has been established, the substrate range of compounds upon which AlkL could act was investigated. It was found that the AlkL protein also enabled activity on tetradecane and hexadecane. The strain containing the pSTBFG plasmid showed only activity on n-octane and no activity on C_12_-C_16_ alkanes, further confirming that AlkL is the necessary component for activity on these longer chain alkanes in *E.coli* (Figure 5).

This supports previous reports that C_12_-C_16_ aliphatic alkanes are viable substrates for this enzyme complex^1^,^2^ and explains the previously observed discrepancy between *in-vivo* activity using *P. putida* Gpo1 pGEc41 (*ΔalkL*) and *in-vitro* activity of the purified enzyme complex^1^.

Using the rhamnose inducible vector co-transformed with pGEc41 also demonstrated initial rate improvements of up to 10-fold with n-octane, up to 120 μmol/min/g and 1 g/L/h (Figure S6). Improved activities on heptane were also observed with rates improving by 75% from 125 mg/L/h to 228 mg/L/h for production of 1-heptanol. With hexane, alkL expression did not improve activity significantly under the conditions tested (110 mg/L/h). Heptadecane oxidation was also attempted which resulted in no detectable C_17_ oxidation products. Since the AlkB enzyme has never before been shown to work on alkanes >C_16_ it remains unclear whether the AlkL protein can act on alkanes longer than hexadecane.

### Activation of the AlkS biosensor

**Figure 6:**
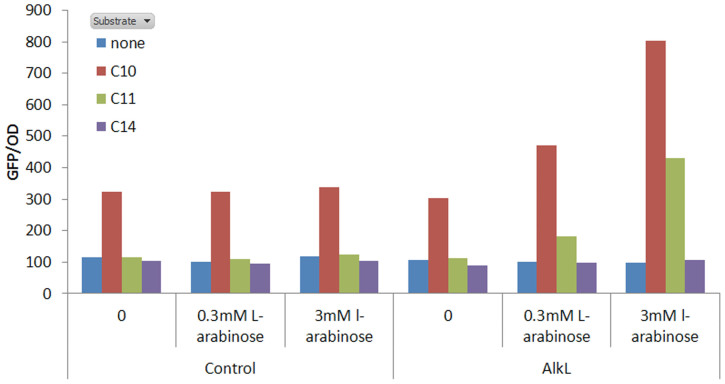
Average specific fluorescence (GFP emission/OD) of *E.coli* containing pSB50C7 (alkS-GFP) by different alkane effector molecules (C10-C14) with and without expression of the alkL protein.

As an alternative method to demonstrate that AlkL facilitates uptake of alkanes, the AlkL protein was co-expressed alongside an alkane biosensor based on the transcription factor AlkS and tested for the rate of uptake of C_10_, C_11_ and C_14_ n-alkanes. Figure 6 demonstrates that as induction of the AlkL protein is increased the rate of specific activation by both C_10_ and C_11_ n-alkanes is increased above the control strain and the strain where alkL is not induced. In the case of decane, an 8-fold increase in specific fluorescence activation was observed when alkL was induced compared to a 3-fold activation in the control cells and uninduced AlkL strains. In the case of undecane no activation was observed without AlkL expression. When AlkL was expressed a 4.5-fold activation was observed with undecane. As expected, no activation was observed with tetradecane as previous studies have shown that AlkS is only activated by alkanes of chain length C_5_ – C_11_^43^. A repeat of the experiment using n-dodecane in place of tetradecane confirmed that dodecane also failed to activate AlkS (data not shown).

### Structural study

**Figure 7:**
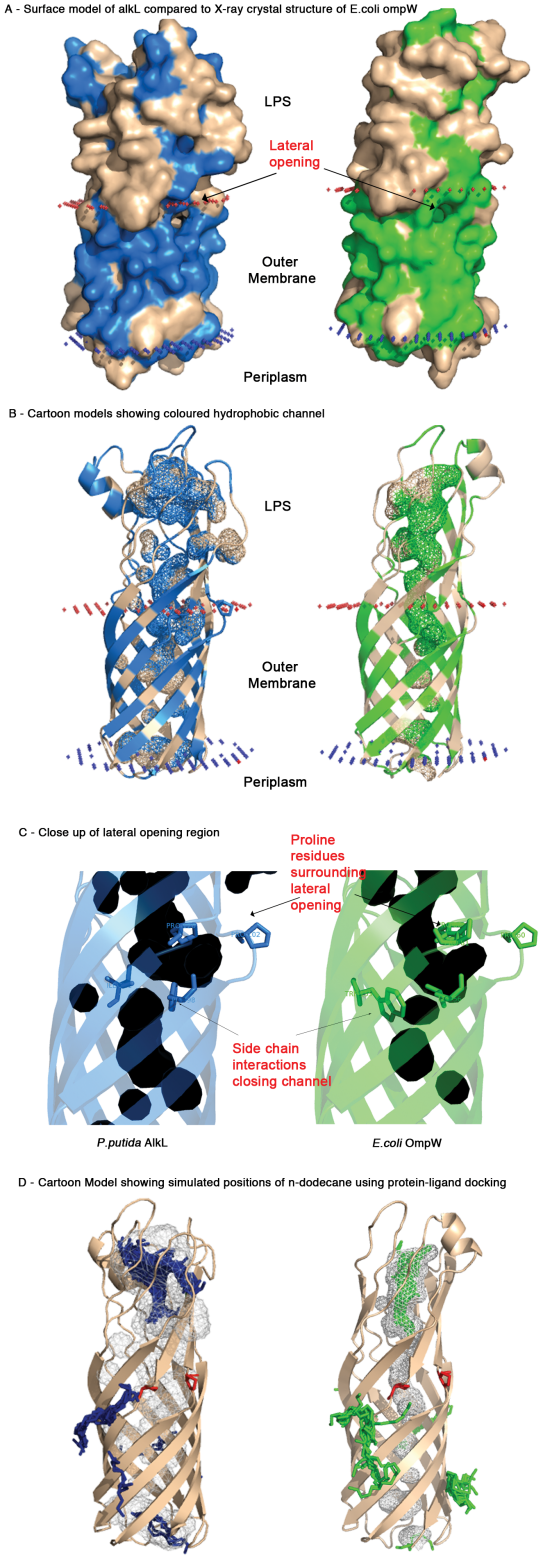
Structural comparison between the homology model of AlkL and the x-ray crystal structure for E.coli OmpW (A) Surface model indicating hydrophobic side chains for AlkL (blue) and OmpW (green) (B) Cartoon structure of AlkL model with cavities shown as a mesh. (C) Close up of the proline residues forming the lateral opening into the outer membrane and the side chain interactions closing the channel beneath the lateral opening. (D) Cartoon structure of AlkL and ompW showing 100 positions of n-dodecane docked using autodock 4.

A homology model of AlkL (Figure 7) was generated by reference to the structures of the *E.coli* OmpW protein (PDB 2f1t). This was the closest template for which an x-ray crystal structure was available (29% sequence identity). The model structure exhibited the same 8 stranded β barrel with a hydrophobic core and the same lateral opening, exiting into the outer membrane. This lateral opening is formed by 2 proline molecules at equivalent positions as found in OmpW; the structural model is therefore consistent with the lateral diffusion hypothesis which is suggested to be the primary mechanism of hydrophobic transport across the cell membrane. The proline molecules surround an opening from the core of the channel into the membrane. The Van den Berg group also proposed there to be a narrowing of the OmpW channel residues which blocks passage through the channel and directs molecules to exit through the lateral opening. In the case of OmpW, side chain interactions between leucine and tryptophan residues on opposite sides of the protein channel seem to act to close the channel beneath the lateral opening (Figure 7). In the AlkL homology model, equivalent side chain interactions are predicted at the same position below the lateral opening but with phenylalanine and isoleucine. The model also indicates that the AlkL protein contains a hydrophobic channel as in OmpW. Docking studies indicated that the predicted region of highest binding energy using n-dodecane as a ligand was observed in an extracellular hydrophobic binding pocket. The equivalent position in the native *E.coli* OmpW protein also bound n-dodecane but the region of predicted binding was narrower (Figure 7D). Due to the seemingly low sequence identity between the OmpW template and AlkL, supplementary homology models were generated using the 2f1tb template and the next closest homologue with an x ray crystal structure, OprG (Pdb 2 × 27) using the Swissmodel, Modweb and Phyre servers. The alignment of the alternative homology models (Figure S5) show some deviation in the extracellular and periplasmic loop regions but the hydrophobic channel and lateral opening are common between the Swissmodel models generated from both the 2f1t template and 2 × 27 templates as well as when the model was generated using the modweb server. Whilst the docking and homology modeling data offer plausible functional explanations for the mechanism of transport through AlkL, this remains speculative at present.

## Discussion

This study provides clear evidence of the role of AlkL in the uptake of medium chain alkanes in the range of C_12_-C_16_. Our findings are complemented by a recent report showing that coexpression of AlkL significantly increased oxygenation of nonane and also dodecanoic acid methyl esters. This supports the evidence of a role in transport hypothesized previously due to the position of the AlkL protein in the alkane degradation operon^23^ and the additional structural parallels between the AlkL protein's hydrophobic core and lateral opening with the fatty acid transporter FadL^31^. The current report now shows that, whilst AlkL is not required for n-octane uptake and subsequent conversion it is necessary for C_12_-C_16_ uptake and subsequent oxidation by the alkane monooxygenase complex. It is therefore likely to play a crucial role in enabling growth of alkane assimilating bacteria on medium-chain alkanes in the wild type *P.putida* GPo1 strain. Further work to mutate the lateral opening residues to block transport would be a useful follow up to demonstrate conclusively that transport does indeed occur directly through this protein rather than as a result of other less likely possibilities such as weakening of the cell membrane as a result of overexpression of the protein. Such lateral opening mutants of the transporter FadL have been used successfully to demonstrate that transport of fatty acids does indeed occur via the lateral opening^20^.

As reported in work by the Van den Berg group, AlkL and OmpW family proteins are widespread in Gram-negative bacteria and yet little is known about the majority of these proteins. The characteristic feature of this particular protein family is the hydrophobic core which is unusual for outer membrane channels which usually contain a hydrophilic core filled with water^21^. The demonstration of the requirement of AlkL in the uptake of C_12_-C_16_ alkanes shown in this paper combined with the hydrophobic channel shared by members of the family hints that uptake of hydrophobic compounds could be the primary function of this protein family. The presence of homologues in operons for the degradation of other hydrophobic molecules such as dibenzothiopene and naphthalene support this hypothesis. The removal of the OmpW outer membrane protein in *Pseudomonas fluorescens* prevented uptake of naphthalene^32^; this is the only other OmpW protein for which the function has so far been proven.

The work also provides further evidence that supports the theory that the outer membrane, and likely the hydrophilic lipopolysaccharide layer, is the principle barrier to uptake of long-chain alkanes in *E.coli*. This has been shown to be the case in the past with lipophilic drugs^41^,^42^,^43^. In the case of methyl dodecanoate, which is structurally similar to dodecane, the addition of the metal chelator EDTA, which is known to disrupt the formation of lipopolysaccharide, has also been shown to improve the permeability of *E.coli* by 2.8-fold^25^.

In conclusion, the work demonstrates that supplementing a whole-cell biocatalyst with an importer active on the substrate of interest can have an enormous impact on the observed activity and should be an important consideration in the engineering design of such systems. The work also demonstrates that when studying the rate of import through a candidate uptake protein, particularly with the intention of building a highly productive whole-cell biocatalyst, the use of a highly active intracellular enzyme can be a particularly effective way to measure the true uptake rate since other assay set-ups such as growth based assays may not reveal the true rate of uptake if uptake is not the rate limiting step.

## Methods

### Material and methods

#### Medium composition

The composition of the aqueous phase in all experiments was either luria broth or as described by Wubbolts et al^30^. KH_2_PO_4_, 4 g/L; K_2_HPO_4_ (3H_2_O), 15.9 g/L; Na_2_HPO_4_ (12H_2_O), 7 g/L; (NH_4_)_2_SO_4_, 1.2 g/L; NH_4_Cl, 0.2 g/L (all from Sigma Aldrich); yeast extract (Oxoid), 5 g/L; L-leucine, 0.6 g/L; L-proline, 0.6 g/L; thiamine, 5 mg/L (All from Alfa Aesar). After autoclaving, MgSO_4_ (7H_2_O) (BDH), 1 g/L (BDH); 1 mL of trace minerals (composition below); 1 mL of 4% (w/v) CaCl_2_(2H_2_O) (Alfa Aesar) and 10 g/L D-glucose (Sigma Aldrich) were added having all been heat sterilized separately. 1 ml of 10 mg/ml filter sterilised tetracycline (Sigma Aldrich) was also added.

The solution of trace minerals contained per liter of 5 M HCL: FeSO_4_ (7H_2_O) (Sigma Aldrich), 40 g; MnSO_4_ (H_2_O) (Sigma Aldrich), 10 g; CoCl_2_ (6H_2_O) (Fluka), 4.75 g; ZnSO_4_ (7H_2_O) (VWR), 2 g; MoO_4_Na_2_ (2H_2_O) (Sigma Aldrich), 2 g; CuCl_2_, (2H_2_O) (Riedel-de Haen), 1 g; H_3_BO_3_ (Sigma Aldrich), 0.50 g.

#### Construction of new plasmids

The *E.coli* GEC137 pGEc47ΔJ strain^33^ contains all components of the OCT plasmid with the exception of the knocked out alcohol dehydrogenase *alkJ*. This was used as a template for PCR amplification of the *alk* genes used to construct the new plasmids.

From the purified pGEc47ΔJ plasmid the *alkST*, *alkBFG* and *alkL* regions were amplified by PCR using the Phusion Hotstart DNA polymerase according to the manufacturers recommended protocol. The primers were designed with a 15 bp overlap which was designed to be complimentary to a 15 bp region at the end of the neighbouring fragment in the desired final construct. In-fusion cloning^27^ was then used to join the fragments together using a 3′ to 5′ exonuclease which reveals the 15 bp overlapping regions which anneal and construct the *alkST*, *alkBFG* and pUC19 fragments into the plasmid in the desired order and orientation.

The *alkL* DNA fragment was introduced into the plasmid by ligating the amplified *alkL* fragment into the TOPO vector and then using restriction digestion to clone from the TOPO-*alkL* vector into the pSTBFG vector. A synthetic palkB promoter was then ligated upstream of the *alkL* to ensure expression under control of the alkS regulator.

The Rhamnose inducible plasmid was constructed from the pRHA67K plasmid^44^ and the *alkL* gene synthesised by DNA 2.0.

The arabinose inducible plasmid was constructed from the pBTB2 plasmid^45^.

pSB50C7 (alkS-GFP) was constructed by amplifying the *alkS* gene and pALKB promoter into pSB50C3 plasmid^46^.

The pSTBFG and pSTBFGL plasmids were purified by the Qiagen miniprep protocol and pGEc47ΔJ was purified by the Qiagen maxiprep protocol. All DNA concentrations were verified by nanodrop and electrophoresis gel. The composition of all plasmids was verified by sequencing.

### Whole-cell bio-oxidation

#### Microwell biotransformation experiments

Carried out in polypropylene 24 deep square well plates with between 1.2 and 2 ml of two-liquid phase medium agitated at 250 rpm with 25 mm throw diameter at 37°C as described previously^44^. The aqueous phase was inoculated with 5% v/v seed culture with 17% v/v organic phase. The medium used is as described under media composition; the organic phase consisted of the respective alkane substrate with 0.05% dicyclopropylketone (DCPK) as inducer of the alk system.

#### Cell density

Cell density measurements were taken by sacrificing the well contents and centrifuging the two-phase samples at 13000 rpm (19000 g) for 15 minutes, marking the aqueous volume on the side of the graduated Eppendorf tube; washing the pellets with tris-HCL pH7.4 and drying in an 80°C oven until a constant mass was reached (24–96 hours).

#### Gas chromatography

Determination of oxidation products in microwells was done via sacrificial sampling and extraction of both phases into ethyl acetate directly. 800 μl of ethyl acetate was added to the two-phase supernatant following centrifugation at 13000 rpm for 5 minutes in a microfuge. The samples were vortexed for 3 × 20 seconds before further centrifugation for 1minute and removal of 100 μl of the organic phase to be analysed by GC-FID as previously mentioned. The proportion of organic phase was determined by cross-referencing the n-alkane peak size to a calibration curve of known standards.

The column used was FID-GC using a SGE BPX5 (30 m long; 0.53 mm internal diameter, 1 μm film) capillary column with helium as carrier gas under a constant pressure of 4PSI. For n-dodecane, n-tetradecane, n-hexadecane oxidation product determination, the samples were eluted at an initial temperature of 150°C for 2minutes, followed by a linear increase of °C minute^−1^ to reach a final temperature of 240°C. Injector and detector temperatures were both 280°C. The concentrations were determined by cross-referencing to a set of the corresponding alkane, 1-alkanol, 1-alkanal and alkanoic acid standards analysed in the same run. For n-octane oxidation product determination the samples were eluted at 70°C followed by a linear increase of 5°C minute^−1^ to reach a final temperature of 145°C. All standards were purchased from Alfa Aesar at the highest purity available (>98%).

#### Design of experiments

Statistical experimental design and interpretation of the results was aided by use of Design Expert 7 from Statease. All experimental designs reported in this paper were general factorial designs.

The comparison of pSTBFG, pSTBFGL and pGEc47ΔJ experiment varied seed culture media (LB or an optimized media described above +10 g/L glucose), antibiotic concentration (100 mg/L or 500 mg/L ampicillin; 4 mg/L or 20 mg/L tetracycline), harvest time (8 hours and 24 hours), substrate (n-octane or n-dodecane), host strain (DH1 or HB101) and plasmid (pSTBFG, pSTBFGL and pGEc47ΔJ) (Table S1). Single colonies transformed with the corresponding plasmid the previous day were used to inoculate 3 ml of seed culture according to the experimental design and 100 μl subcultured after 11 hours into 900 μl fresh media containing the antibiotic concentration set out in the experimental design and 200 μl of the alkane substrate, 4% v/v Dimethyl sulphoxide (DMSO), 0.1% v/v triton-x-100 and 0.05% v/v dicyclopropylketone (DCPK).

The substrate range experiment varied plasmid (pSTBFG and pSTBFGL), substrate (n-octane, n-dodecane, n-tetradecane and n-hexadecane) and harvest time (8 hours and 20 hours). The experimental protocol was the same as above but with the following fixed factors: the host strain was DH1; the seed medium (see composition above) contained 10 g/L glucose; the antibiotic concentration was 500 mg/L ampicillin.

Assays using the pGEc41 plasmid cotransformed with the pRha67K *alkL* vector were performed in HB101 and used the protocol as described above without DMSO addition and using 10 g/L glycerol in place of glucose as the carbon source so that glucose repression of the RhaR RhaS system did not inhibit AlkL expression. The dodecane oxidation study was harvested after 24 hours and the substrate screening experiment pre-induced the AlkB and AlkL expression for 4 hours prior to substrate addition and the reaction was quenched and harvested after 1 hour by addition of ice-cold ethyl acetate.

#### Structural modeling

Structural alignment and viewing the structure of AlkL and OmpW was performed using Pymol. The models were generated using Swiss Model^34^,^35^ using the 2f1t x-ray crystal structure as a template. The membrane position was predicted using the orientations of proteins in membranes database^36^

#### SDS-PAGE AlkB expression determination

Lysate samples were prepared by resuspending cell pellets in distilled water and sonicating for 10 × 10 s pulses with 10 s between each pulse. The Bradford assay^37^ was then used to determine protein concentration before loading standardised protein concentrations of 5 μg per lane. SDS-PAGE^38^ was then carried out according to the Invitrogen Nupage method^39^. Densitometry was used to compare band intensity as a % of the whole lane after background intensity was subtracted using the rolling disk method.

#### Western blot

To confirm expression, a C-terminal 6HIS *alkL* construct was synthesised which expressed *alkL* from a second plasmid under control of the rhamnose promoter and under kanamycin resistance. This was used to transform and complement the pGEC41 (*ΔalkL*) construct to restore the dodecane oxidation phenotype. HB101 Cells were cultured overnight and induced with 10 mM Rhamnose and harvested after 24 hours. SDS-PAGE was performed as described above loading 10 μg of protein per lane. Semi-dry transfer was performed after thoroughly washing the gel in phosphate-bufffered saline. Blocking was performed using Amresco's rabidblock™ solution followed by incubation overnight at 4°C in the anti-HIS rabbit antibody with near infrared emitting dye conjugate. The blot was then washed thoroughly in PBS + 0.01% Tween and analysed using the Licor Odyssey^40^.

#### Alkane biosensor assay

*E.coli* DH5αZ1 and *E.coli* HB101 were transformed with an alkS-Superfolder GFP biosensor and cotransformed with either (i) a control expression vector pBTB2 containing no gene or (ii) pBTB2 containing *alkL*. The strains were grown up overnight from single colonies in parallel in 24 Deep square well plates (1 ml per well) at 37°C and 250 rpm (25 mm throw diameter) in Luria broth containing 5 g/L glucose and then sub cultured (4% incoculum) into the media described above containing 10 g/L glycerol, grown for 2 hours, induced using either 0, 0.3 mM or 3 mM L-arabinose as appropriate. After 3 hours growth, 1% v/v alkane substrates were added and grown for 3 hours before analysing fluorescence (excitation 485 nm; emission 517 nm) and OD using the BMG Clariostar plate reader.

## Supplementary Material

Supplementary Information Supplementary information
