# Supplementary material for: Identification and use of an alkane transporter plug-in for applications in biocatalysis and whole-cell biosensing of alkanes
Source: Sci Rep. 2014 Jul 28;4:5844. doi: 10.1038/srep05844 (PMC5376172; doi:10.1038/srep05844)
Supplement: Supplementary Information [file srep05844-s1.doc]

**Supplementary Information**

**Identification and use of an alkane transporter plug-in for applications in biocatalysis and whole-cell biosensing of alkanes**

Chris Grant^1^, Dawid Deszcz^1^, Yu-Chia Wei^1^, Rubens Julio Martinez-Torres^2^, Phattaraporn Morris^1^, Thomas Folliard^1^, Rakesh Sreenivasan^1^, John Ward^1,2^, Paul Dalby^1^, John M Woodley^3^, Frank Baganz^1^

^1^ Dept. of Biochemical Engineering, Advanced Centre for Biochemical Engineering, University College London, Torrington Place, London WC1E 7JE, U.K.

^2^ Dept. of Structural and Molecular Biology, ISMB, University College London, Gower Street, London WC1E 6BT, U.K.

^3^ Department of Chemical and Biochemical Engineering, Technical University of Denmark, DK 2800 Lyngby, Denmark

**Table S1 Factorial design for testing the function of the AlkL outer membrane protein and identifying the most stable conditions for activity in an unstable high copy number plasmid.**

|  | Factor | Rationale | Level 1 | Level 2 | Level 3 |
| --- | --- | --- | --- | --- | --- |
| A | Host Strain | Improve host stability for expression of AlkB/AlkL with HB101^17^ | HB101 | DH1 |  |
| B | Plasmid | +ve control for alkane hydroxylase activity; +/- control for AlkL function | pSTBFG | pSTBFGL | pGEC47ΔJ |
| C | [Antibiotic] | Improve plasmid stability ^41^ | 1X | 5X |  |
| D | Seed Media | Repress Lac expression with glucose ^42^ | -Glucose | +Glucose |  |
| E | T_Incubation_ | Is product metabolism/expressional stability an issue? | 8h | 20h |  |
| F | Substrate | Is a functional alkane hydroxylase being expressed? | N-octane | N-dodecane |  |


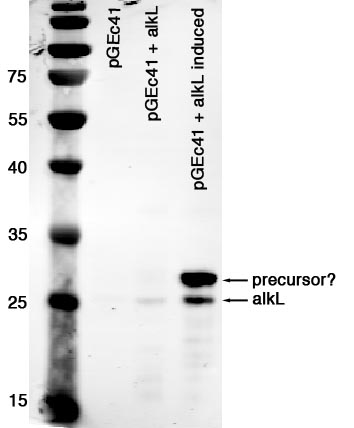


Figure S1 Western blot showing alkL expression in *E.coli* HB101 without induction and 24 hours after induction according to the description in the material and methods section. It was found that the leaky expression of alkL was sufficient to enable n-dodecane oxidation.


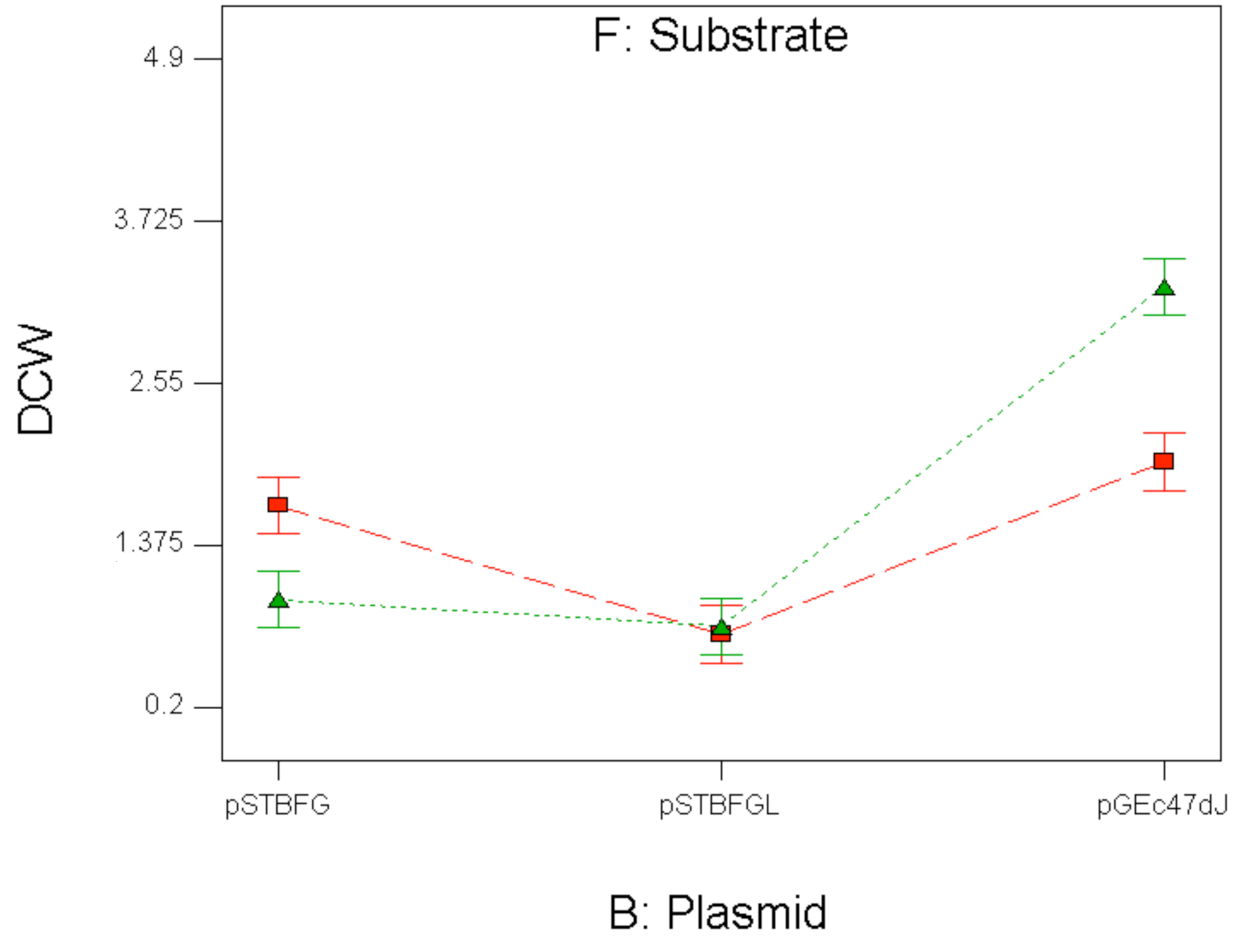


Figure S2 Average dry cell weight of *E. coli* HB101? growth on n-octane (red squares) and n-dodecane (green triangles) containing the pSTBFG, pSTBFGL or pGec47ΔJ plasmids. Error bars represent the least significant difference.

**
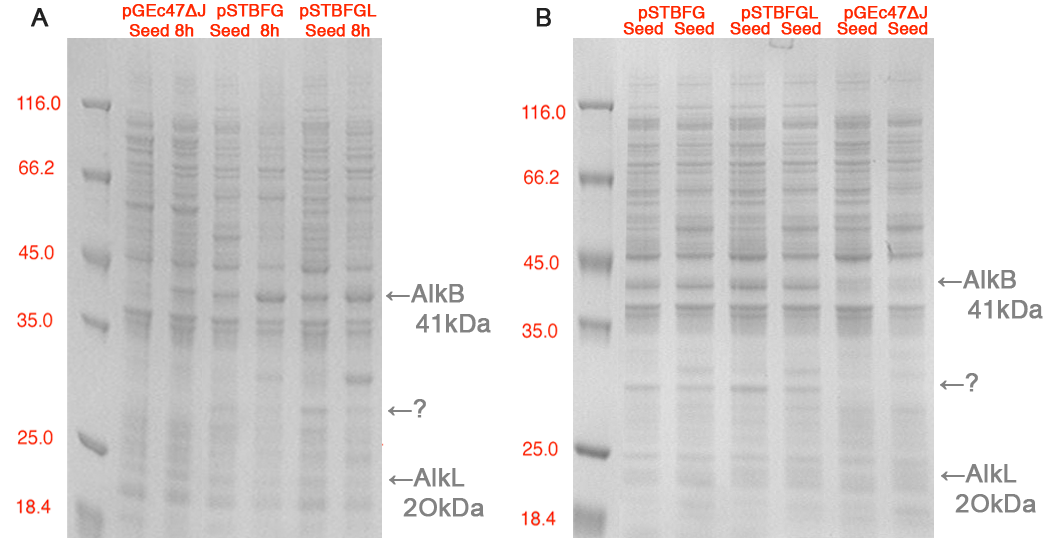
**

Figure S3 SDS PAGE gels showing (A) pre-induction and 8h post induction lysate of the three plasmids (B) The pre-induction cell lysates of the three plasmids in *E.coli* DH1.


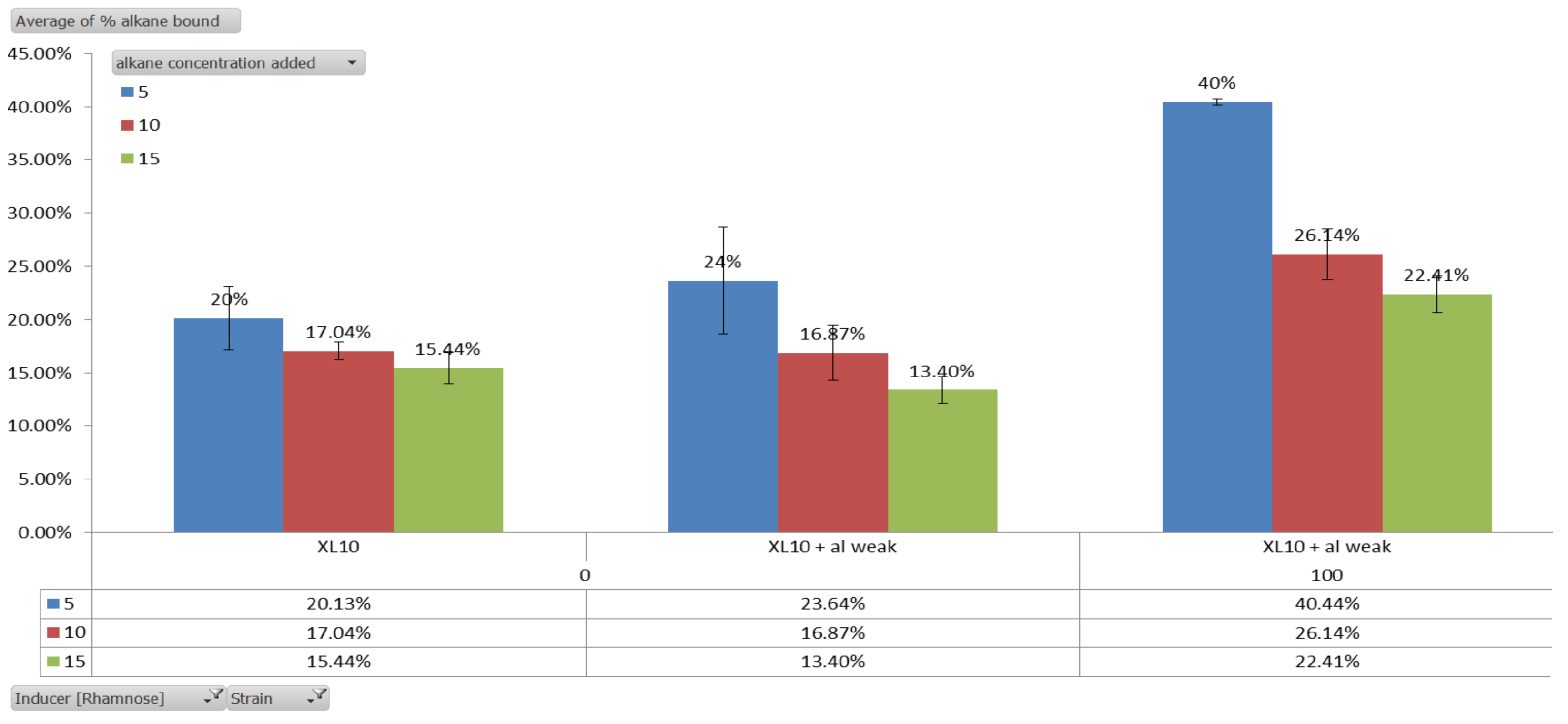


**Figure S4** Effect of alkL induction (al weak) of 0 and 100μM L-Rhamnose on intracellular alkane concentration after incubation with dodecane compared to a control of *E.coli* XL10.


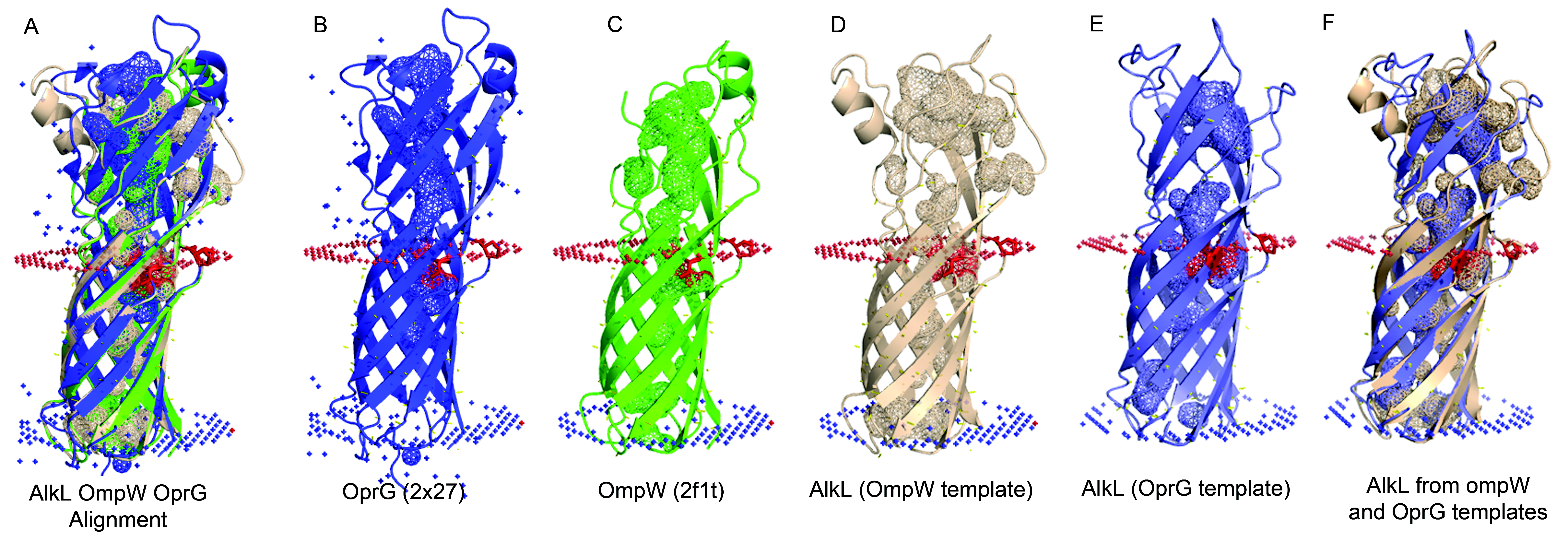


**Figure S5** structural similarities between homology models of alkL generated by different templates


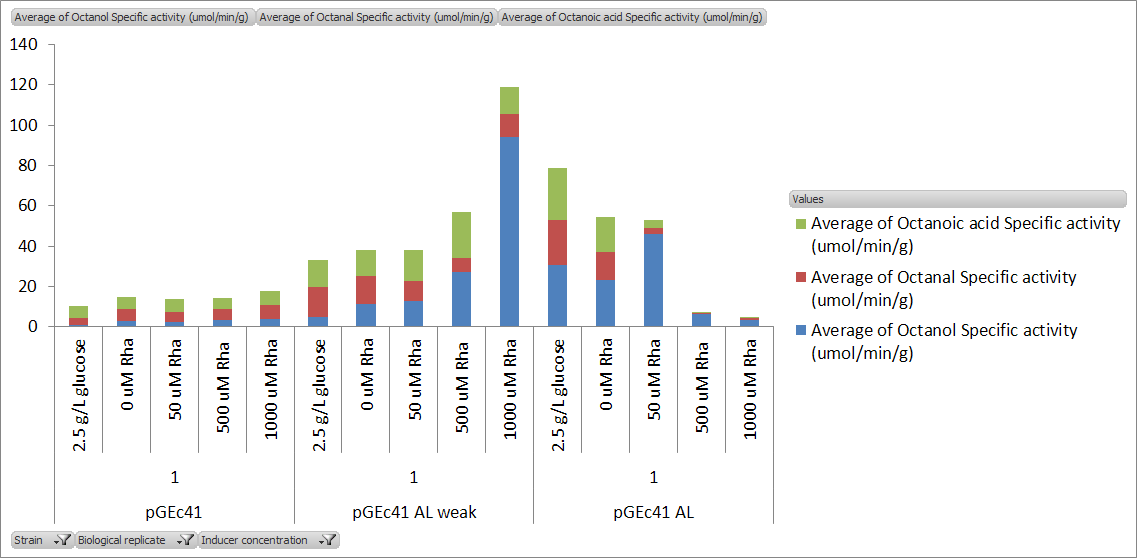


**Figure S6** *Effect of alkL expression level on specific activities for octane oxidation. Graphs show the change in oxidation products of the n-octane bio-oxidation using alkB, alkG and alkT from the pGEc41 plasmid by co-expressing alkL using either a low-range or high range expression plasmid under different inducer concentrations. The Y-axis shows the specific activity calculated after 1 hour compared to the negative control with only the pGEc41 plasmid. The numbers on the x-axis represent inducer concentrations in μM of Rhamnose.*
